# Recombinant thrombomodulin prevents acute lung injury induced by renal ischemia-reperfusion injury

**DOI:** 10.1038/s41598-019-57205-0

**Published:** 2020-01-14

**Authors:** Naoki Hayase, Kent Doi, Takahiro Hiruma, Ryo Matsuura, Yoshifumi Hamasaki, Eisei Noiri, Masaomi Nangaku, Naoto Morimura

**Affiliations:** 1 Department of Acute Medicine, The University of Tokyo, 7-3-1 Hongo, Bunkyo-ku, Tokyo 113-0033 Japan; 2 Department of Nephrology and Endocrinology, The University of Tokyo, 7-3-1 Hongo, Bunkyo-ku, Tokyo 113-0033 Japan

**Keywords:** Acute kidney injury, Experimental models of disease, Innate immunity

## Abstract

Acute kidney injury (AKI) complicated by acute lung injury has a detrimental effect on mortality among critically ill patients. Recently, a renal ischemia-reperfusion (IR) model suggested the involvement of histones and neutrophil extracellular traps (NETs) in the development of distant lung injury after renal IR. Given that recombinant thrombomodulin (rTM) has anti-inflammatory roles by binding to circulating histones, we aimed to clarify its effect on distant lung injury induced by AKI in a murine bilateral renal IR model. Both pretreatment and delayed treatment with rTM significantly decreased pulmonary myeloperoxidase activity, but they did not affect renal dysfunction at 24 h after renal IR. Additionally, rTM mitigated the renal IR-augmented expression of proinflammatory cytokines (tumor necrosis factor-α, interleukin-6, and keratinocyte-derived chemokine), and vascular leakage, as well as the degree of lung damage. Intense histone accumulation and active NET formation occurred in both the kidneys and the lungs; however, rTM significantly decreased the histone and NET accumulation only in the lungs. Administration of rTM may have protective impact on the lungs after renal IR by blocking histone and NET accumulation in the lungs, although no protection was observed in the kidneys. Treatment with rTM may be an adjuvant strategy to attenuate distant lung injury complicating AKI.

## Introduction

Acute kidney injury (AKI) complicated by multiple organ failure places a severe burden on critically ill patients and is associated with a mortality rate that exceeds 80%^1,2^. There is increasing evidence that the kidneys interact with distant organs, including the lungs, heart, liver, and intestines in these patients^3–6^. Despite of better understanding of organ cross-talk in AKI, no therapeutic strategy has been developed to prevent and treat the AKI-induced distant organ injury.

The interaction between the kidneys and lungs is well-documented. Among the distant organ failures that occur during AKI, clinical research has shown that respiratory failure is most strongly associated with in-hospital mortality in patients with AKI requiring hemodialysis^1^. Experimental studies with bilateral nephrectomy and ischemia-reperfusion (IR) injury have also revealed the involvement of several factors in the pathogenesis of distant lung injury that is characterized by neutrophil infiltration. These include circulating interleukin (IL)-6 and pulmonary chemokine (C-X-C motif) ligand 1 (CXCL1) expression^7,8^, high morbidity group box-1 (HMGB1) and toll-like receptor 4 (TLR 4) activation^9^, and histones that promote neutrophil extracellular traps (NETs)^10^.

NETs were first identified by Brinkmann *et al*. as an innate immune response of neutrophils against various pathogens^11^. However, existing evidence suggested that, in addition to their bactericidal activity, these netting neutrophils exerted cytotoxic effects on bystander host cells by releasing histones and proteases into the extracellular matrix. Histones further activate neutrophils via TLRs (TLR 2, 4, and 9) to form NETs, resulting in an auto-amplification of histones^12,13^. Recently, Nakazawa *et al*. showed that NET formation and NET-derived histones could not only promote tubular epithelial cell death but also induce distant lung injury in bilateral renal IR injury^10^.

Thrombomodulin (TM) is a transmembrane glycoprotein expressed mainly on the endothelium of blood vessels, where it catalyzes protein C activation and exerts an anticoagulant activity when complexed with thrombin^14^. Additionally, the N-terminal lectin-like domain of TM has an anti-inflammatory function that is exerted through interaction with HMGB1^15^. The soluble form of TM has been shown to attenuate ischemic kidney injury in a hypoperfusion model of renal ischemia^16^. Moreover, the recent basic study reported that recombinant TM (rTM) could inhibit histone-induced lethal thromboembolism by binding to circulating histones in histone-challenged mice^17^. To date, however, it remains unknown whether rTM can protect against remote organ injury in AKI.

We aimed to elucidate the effect and mechanism of rTM on distant lung injury induced by AKI in a murine bilateral renal IR model. We also studied the beneficial effects of rTM in the context of local kidney injury.

## Results

### Lung myeloperoxidase (MPO) activity and renal function

**Figure 1:**
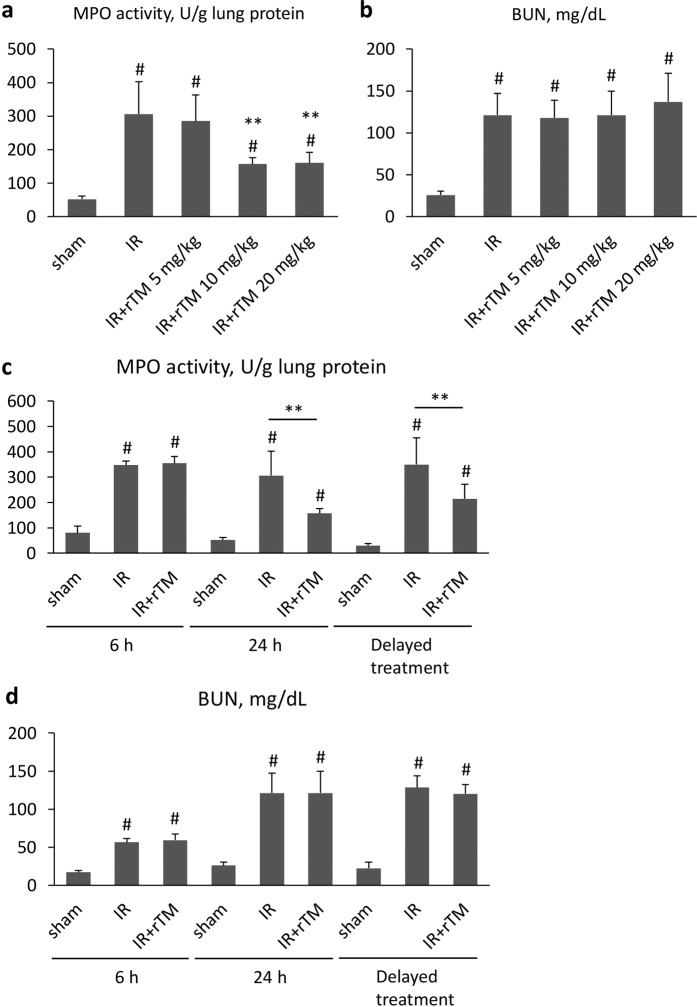
Lung MPO activity and plasma BUN after AKI induced by IR insults. The association of rTM treatment with lung MPO activity and renal function was evaluated after ischemic AKI induced by bilateral renal pedicle clamping for 30 min. (**a**) Pretreatment dosage of rTM and lung MPO activity at 24 h after surgery (n = 4 in sham group, n = 6 in IR and IR + rTM groups). (**b**) Pretreatment dosage of rTM and plasma BUN elevation at 24 h after IR insults (n = 4 in sham group, n = 6 in IR and IR + rTM groups). (**c**) Effect of pretreatment with 10 mg/kg rTM on lung MPO activity in mice at 6 h and 24 h after surgery (n = 5 in 6 h group, n = 6 in 24 h group) and effect of delayed treatment (administration of 10 mg/kg rTM at 6 h after renal IR) at 24 h after surgery (n = 5 in delayed treatment group). (**d**) Effect of pretreatment with 10 mg/kg rTM on plasma BUN levels in the mice at 6 h and 24 h after renal IR (n = 5 in 6 h group, n = 6 in 24 h group) and effect of delayed treatment at 24 h after surgery (n = 5 in delayed treatment group). **P < 0.05 versus the IR group, ^#^P < 0.05 versus sham. Abbreviations: AKI, acute kidney injury; BUN, blood urea nitrogen; IR, ischemia-reperfusion; MPO, myeloperoxidase; rTM, recombinant thrombomodulin.

Firstly, we measured MPO activity in the lung tissue and plasma blood urea nitrogen (BUN) level at 24 h after surgery, based on the previous finding that marked increase of neutrophil margination was observed in the lung at 24 h following renal IR^3^. Pulmonary MPO activity and plasma BUN levels were significantly increased after renal IR compared with sham-operated animals. Pretreatments with 10 and 20 mg/kg of rTM (but not 5 mg/kg) markedly attenuated the increased MPO activity in the lung, but had no impact on BUN levels (Fig. 1a,b). When we further compared the effect of pretreatment with 10 mg/kg rTM on lung and kidney injury between 6 h and 24 h after renal IR, a beneficial effect on lung MPO activity was only detected at 24 h. There was no significant difference in BUN levels between the IR groups with and without rTM at either time point (Fig. 1c,d). Additionally, delayed treatment at 6 h after renal IR (10 mg/kg) also suppressed lung MPO activity, not BUN elevation, at 24 h following surgery (Fig. 1c,d).

### Lung and kidney histology

**Figure 2:**
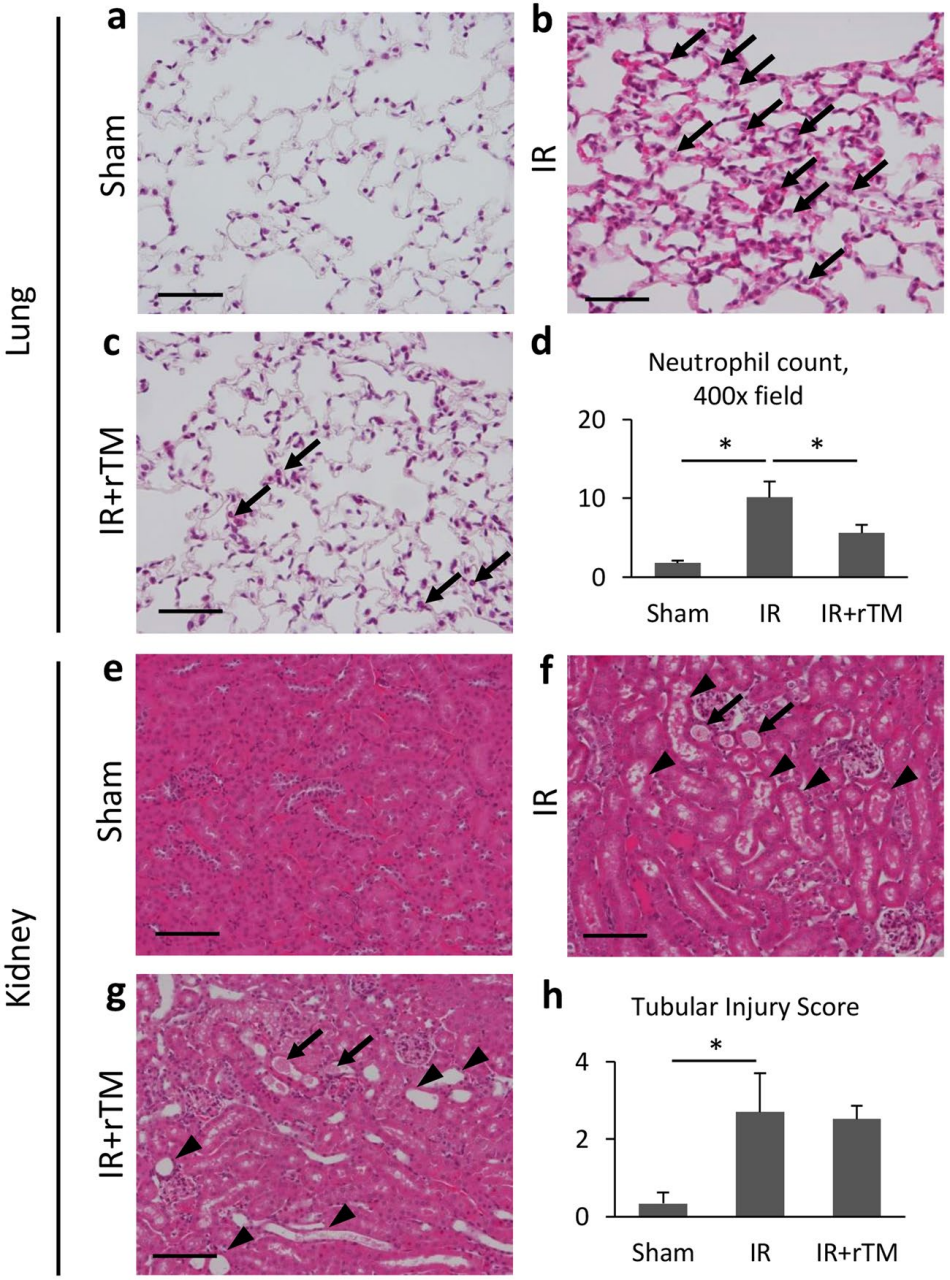
Pulmonary and renal histology of renal IR-injured mice. The mice were injected with 10 mg/kg of rTM or vehicle solution at 30 min before surgery and subjected to bilateral renal pedicles clamping for 30 min followed by 24-h reperfusion. Representative images of lung sections (hematoxylin and eosin staining) from (**a**) the sham-operated, (**b**) IR, and (**c**) IR + rTM groups are shown. Scale bar = 50 µm. Pulmonary histology after renal IR is characterized by septal edema and neutrophil infiltration (arrows). (**d**) The number of neutrophils in the lung after surgery (n = 5 per group). Representative image of renal sections (hematoxylin and eosin staining) from (**e**) the sham-operated, (**f**) IR, and (**g**) IR + rTM groups are shown. Scale bar = 50 µm. Arrows (**f**,**g**) denote cast formation, and arrowheads (f and g) exhibit tubular dilation and loss of brush border. (**h**) Average score of tubular injury for respective animals was determined. n = 5 per group. *P < 0.05 versus respective control. Abbreviations: IR, ischemia-reperfusion; rTM, recombinant thrombomodulin.

Histological analyses demonstrated significant neutrophil infiltration and septal edema in the lung at 24 h following renal IR (Fig. 2b). Pretreatment with 10 mg/kg rTM appeared to suppress this neutrophil recruitment in the lung (Fig. 2c). Indeed, rTM significantly attenuated the increased number of neutrophils in the lung after ischemic AKI (Fig. 2d). Necrotic tubular injury, characterized by loss of the brush border, tubular dilation, and cast formation, was observed in the kidneys following the IR injury (Fig. 2f), but there was no significant difference in renal histology between groups that did and did not receive rTM (Fig. 2g). Quantification with the tubular injury score revealed that rTM failed to ameliorate the IR-induced kidney injury (Fig. 2h). Moreover, delayed treatment with rTM showed the similar impact on renal IR-induced histological damage in the lungs and kidneys (See Supplementary Fig. S1).

### Cytokine expression

**Figure 3:**
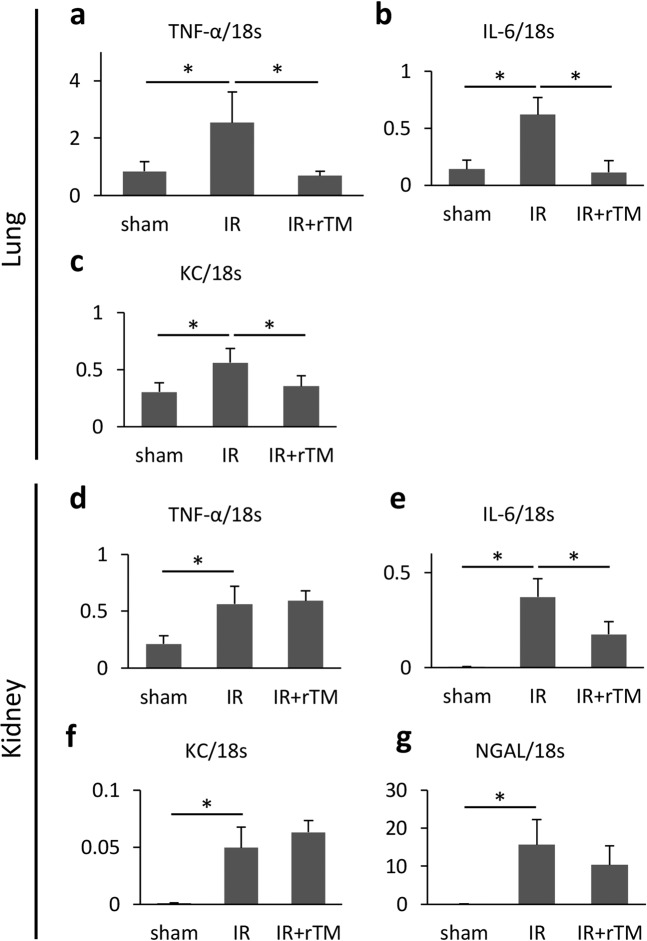
Proinflammatory gene mRNA expression in the lungs and kidneys after renal IR. Bilateral renal IR model mice (30-min ischemia followed by 24 hours of reperfusion) were pretreated with saline (IR group) or 10 mg/kg of rTM (IR + rTM group). Pulmonary mRNA expressions of (**a**) TNF-α, (**b**) IL-6, and (**c**) KC were shown. The mRNA expressions of (**d**) TNF-α, (**e**) IL-6, (**f**) KC, and (**g**) NGAL were also evaluated in the IR-injured kidney. n = 5 per group. *P < 0.05 versus respective control. Abbreviations: IL, interleukin; IR, ischemia-reperfusion; KC, keratinocyte-derived chemokine; mRNA, messenger RNA; NGAL, neutrophil gelatinase-associated lipocalin; rTM, recombinant thrombomodulin; TNF-α, tumor necrosis factor-α.

To assess the effect of rTM on the inflammatory response in the local and distant organs during ischemic AKI, we measured the messenger RNA (mRNA) expression levels of inflammatory cytokines including tumor necrosis factor-α (TNF-α), keratinocyte-derived chemokine (KC), and IL-6 in the lung and kidney using quantitative polymerase chain reaction (PCR). Renal IR increased pulmonary gene expressions of TNF-α, KC, and IL-6, while pretreatment with rTM decreased the expressions of these cytokines in the lung (Fig. 3a–c). However, rTM failed to suppress the increased expression of inflammatory genes in the kidney, except for IL-6 (Fig. 3d–f), and did not significantly reduce the IR-augmented expression of neutrophil gelatinase-associated lipocalin (NGAL) (Fig. 3g).

### Plasma cytokine levels

Plasma levels of IL-1β, IL-2, IL-6, TNF-α, and IL-10 were measured after 24 h to examine how rTM affected the systemic inflammatory response after renal IR. Renal IR increased the level of each cytokine remarkably, but pretreatment with rTM significantly reduced TNF-α, IL-6, and IL-10 levels. The levels of IL-1β and IL-2 also fell with rTM pretreatment when compared with no treatment, but without statistical significance (See Supplementary Fig. S2).

### Vascular permeability

**Figure 4:**
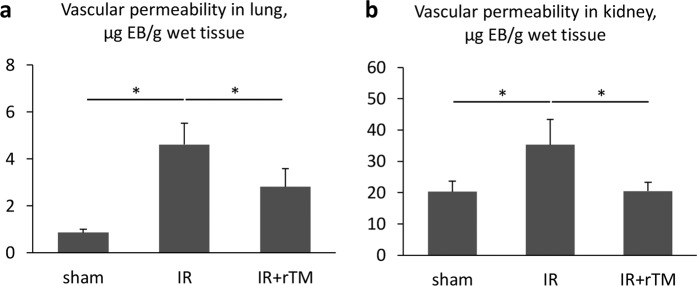
Vascular permeability in the lungs and kidneys after bilateral renal IR. EBD contents were determined in the lung and kidney at 24 h after bilateral renal IR. The mice were pretreated with saline (IR group) or 10 mg/kg of rTM (IR + rTM group). n = 4 in sham group; n = 6 in IR and IR + rTM groups. *P < 0.05 versus respective control. Abbreviations: EBD, Evans blue dye; IR, ischemia-reperfusion; rTM, recombinant thrombomodulin.

Next, pulmonary and renal vascular permeability was examined by Evans blue dye (EBD) assay at 24 h to assess the contribution of rTM pretreatment to vascular leakage after renal IR. We found that vascular leakage increased significantly in the lungs and kidneys after renal IR injury, but that this vascular permeability was reduced in both organs by pretreatment with rTM (Fig. 4a,b).

### Histone accumulation and NET formation

**Figure 5:**
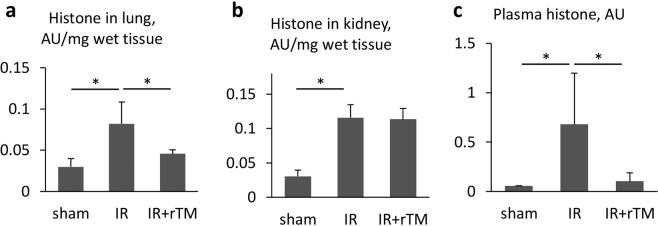
Accumulation of histones in the lung and kidney after renal IR. Histone levels in (**a**) the lung, (**b**) kidney, (**c**) plasma were evaluated after 30-min ischemia followed by 24 -h reperfusion. The mice were pretreated with saline (IR group) or 10 mg/kg of rTM (IR + rTM group). n = 6 per group. *P < 0.05 versus respective control. Abbreviations: AU, absorbance unit; IR, ischemia-reperfusion; rTM, recombinant thrombomodulin.

Reportedly, the largest amount of citrullinated histone H3 (CitH3), which is associated with NET formation, was detected in the lung and kidney among organs during ischemic AKI^10^. We therefore sought to determine the effect of rTM on the histone content of these organs at 24 h after renal IR. Pretreatment with rTM markedly decreased renal IR-augmented histone levels in the lung, but did not significantly reduce histone levels in the IR-injured kidney (Fig. 5a,b). Moreover, plasma histone levels were prominently elevated in the ischemic AKI mice compared with the sham-treated mice. Pretreatment with rTM was associated with a significant decrease in histone levels in the plasma of mice following renal IR (Fig. 5c). Similarly, delayed treatment with rTM significantly attenuated renal IR-augmented histone accumulation in the lung and plasma, not in the kidney (See Supplementary Fig. S3).

**Figure 6:**
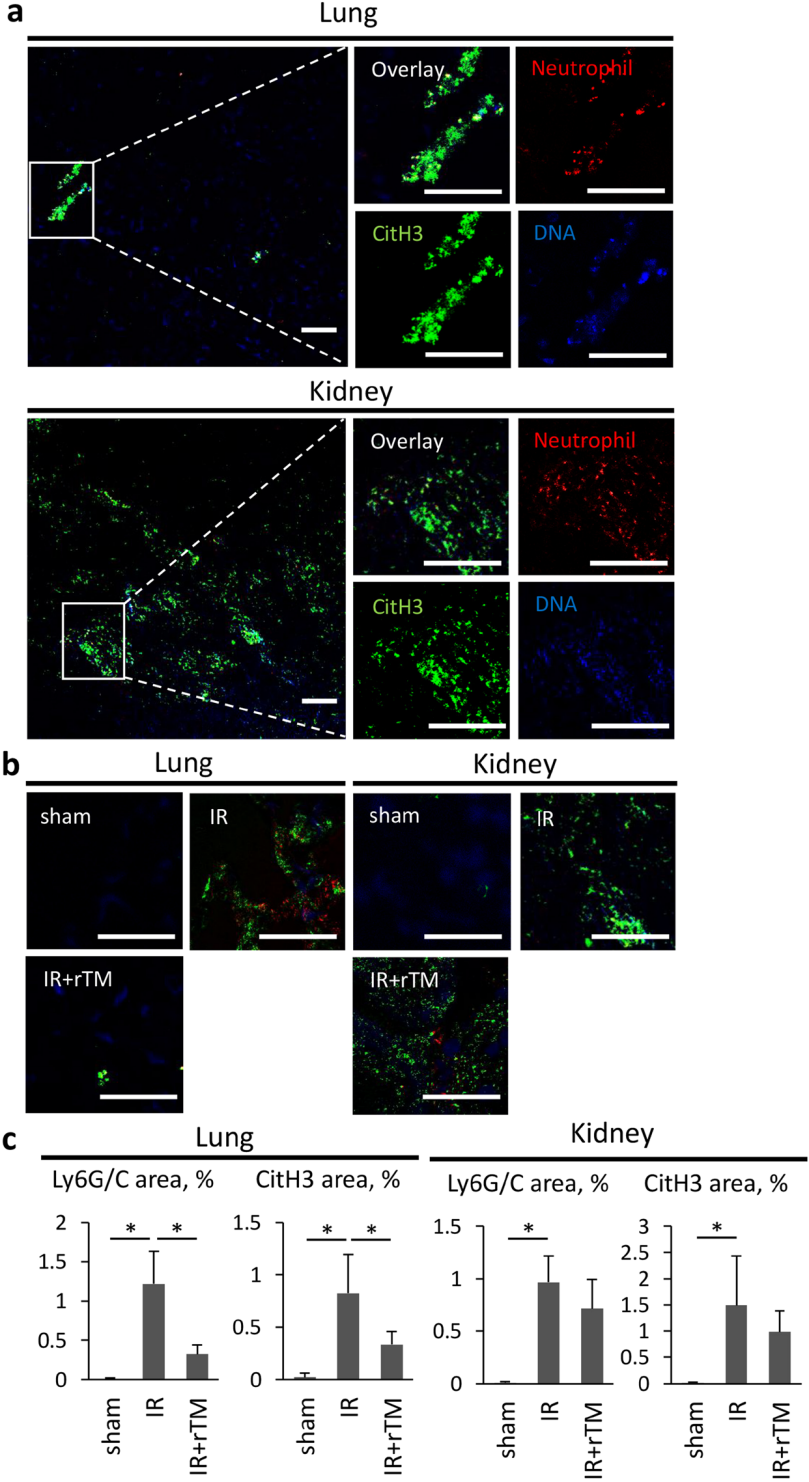
NET formation in the lungs and kidneys after renal IR. Bilateral renal IR model mice (30-min ischemia followed by 24 hours of reperfusion) were pretreated with saline (IR group) or 10 mg/kg of rTM (IR + rTM group). (**a**) Representative images of NETs in the lung and kidney are shown with positive staining for CitH3 (green), Ly6G/C (red), and DNA (blue). The colocalization of CitH3, Ly6G/C and DNA indicates NET formation. The impact of rTM pretreatment on NET formation in the lung and kidney were assessed by (**b**) an immunofluorescence assay and (**c**) quantitative analysis of NETs at 24 h after bilateral renal IR. Scale bar, 25 µm. n = 7 per group. *P < 0.05 versus respective control. Abbreviations: CitH3, citrullinated histone H3; IR, ischemia-reperfusion; NET, neutrophil extracellular trap; rTM, recombinant thrombomodulin.

We further evaluated whether NET formation was consistent with the increased abundance of histone in the kidneys and lungs, using immunofluorescence staining and quantitative analysis. At 24 h after renal IR, we observed Ly6G/C-positive leukocytes colocalizing with CitH3 and positively stained DNA in the lungs and kidneys, suggesting that NET formation had occurred (Fig. 6a). Consistent with the findings of histone quantification, the amount of NETs was lower in the lungs of mice pretreated with rTM, whereas NET levels were comparable in the kidneys of treated and untreated mice (Fig. 6b,c). Furthermore, delayed treatment with rTM also reduced the amount of NETs augmented by renal IR in the lungs, but not in the kidneys (See Supplementary Fig. S4).

**Figure 7:**
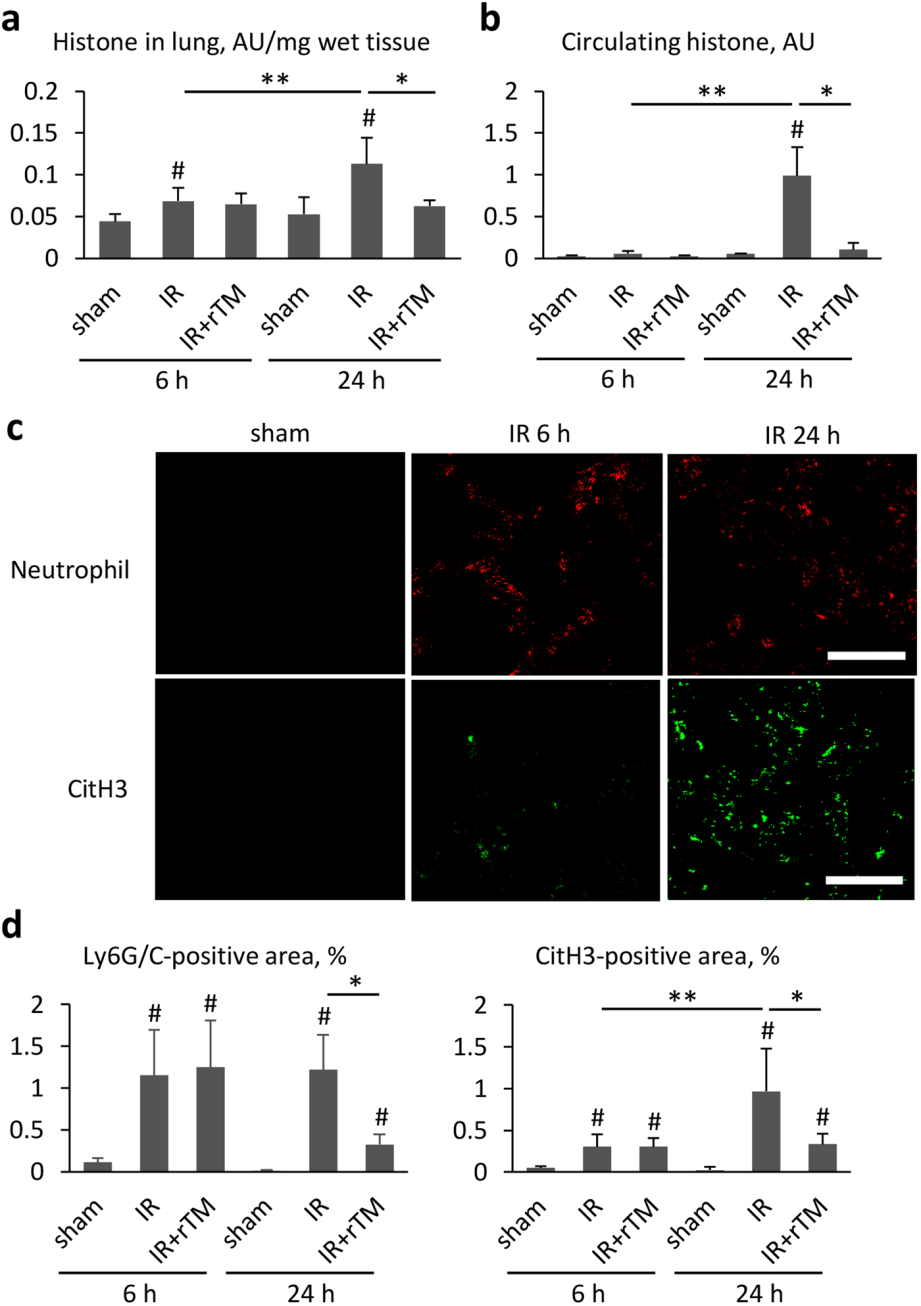
Histone levels in the lungs and plasma after renal IR. The effect of renal IR and rTM pretreatment on histone contents in (**a**) the lung and (**b**) plasma were evaluated at 6 h and 24 h following bilateral renal IR. (n = 6 per group) (**c**) Pulmonary neutrophils and histones at 6 h and 24 h after bilateral renal IR were exhibited by immunostaining of Ly6G/C (neutrophil marker) and CitH3. (**d**) Ly6G/C- and CitH3-positive area were also calculated at 6 h and 24 h after surgery. Scale bar, 25 µm. n = 7 per group. ^#^P < 0.05 versus sham. *P < 0.05 between the IR and IR + rTM group. **P < 0.05 between 6 h and 24 h. Abbreviations: AU, absorbance unit; CitH3, citrullinated histone H3; IR, ischemia-reperfusion; rTM, recombinant thrombomodulin.

When we compared pulmonary and plasma histone levels at 6 h and 24 h after renal IR, both were significantly higher at 24 h. Pretreatment with rTM resulted in a marked decrease in lung and plasma histone levels at 24 h; however, there were no significant reductions in histone levels at 6 h between the treated and untreated groups (Fig. 7a,b). Consistent with the results of the MPO activity assay, immunofluorescence staining and quantitative analysis showed numerous Ly6G/C-positive cells, which indicated neutrophil infiltration, appeared in the lung as early as 6 h, whereas CitH3 production (indicating active NET formation) increased significantly at 24 h compared to at 6 h (Fig. 7c,d).

### Plasma HMGB1 levels

**Figure 8:**
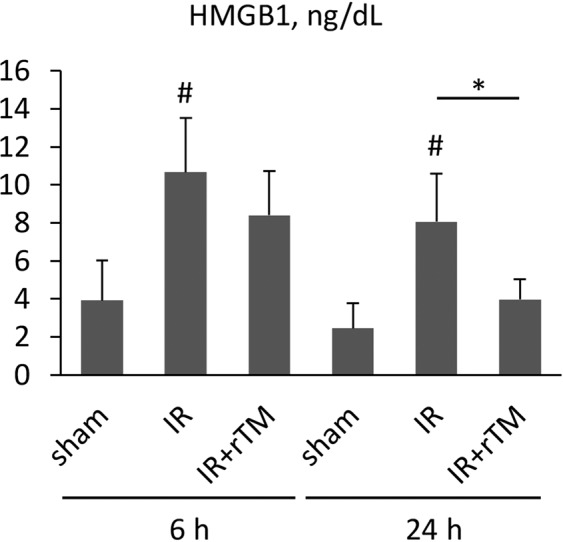
Plasma HMGB1 levels in mice after renal IR. The plasma levels of HMGB1 were measured at 6 h and 24 h after bilateral renal IR. The mice were pretreated with saline (IR group) or 10 mg/kg of rTM (IR + rTM group). n = 6 per group. *P < 0.05 versus respective control. Abbreviations: HMGB1, high morbidity group box-1; IR, ischemia-reperfusion; rTM, recombinant thrombomodulin.

A previous report showed that HMGB1 contributed to renal IR-induced lung injury by recruiting activated neutrophils^9^. Because rTM elicits an anti-inflammatory function by binding to HMGB1, we assessed whether pretreatment with rTM affected the elevated plasma levels of HMGB1 in mice after renal IR. Plasma HMGB1 was markedly increased at 6 h after renal IR, and this elevation persisted for 24 h. Although there were no significant differences in HMGB1 levels between the treated and untreated groups at 6 h, there was a significant reduction in HMGB1 at 24 h in the group that received rTM (Fig. 8).

## Discussion

There is a growing body of evidence that AKI complicated by multiple organ dysfunction is associated with increased mortality in critically ill patients^1,2^. A recent study suggested that extracellular histones may have a crucial role in the development of distant organ injury in AKI by promoting NET formation^10^. In Japan, rTM is widely used in disseminated intravascular coagulopathy, and it has been recognized to have anti-inflammatory properties by sequestering damage-associated molecular patterns, such as extracellular histones and HMGB1^15,17^. We report that both pretreatment and delayed treatment with rTM effectively suppressed distant lung injury in a murine renal IR model by inhibiting histone accumulation and NET formation in the lungs without affecting local kidney injury.

Several mechanisms of cross-talk between the kidneys and lungs have been proposed, including dysfunction of water clearance, activation of neutrophils with the secretion of neutrophil elastase, circulating IL-6 and pulmonary CXCL1 expression, and the activation of TLR 4 by plasma HMGB1, and NETs and NET-derived histones^7–10,18,19^. In our study, intense histone accumulation and active NET formation occurred in both the kidneys and the lungs of mice with IR-induced AKI, as previously described^10^. Whereas abundant neutrophil infiltration was evident in the lungs from 6 h after renal IR according to the MPO activity (Fig. 1c) and immunofluorescence results (Fig. 7c,d), pulmonary and plasma histone levels were significantly higher at 24 h than at 6 h (Fig. 7a–d). This time-dependent increase in histones may suggest that their release from damaged tubular cells induces neutrophils to trigger NET formation, which leads to auto-amplification loop of histone production. Further evaluation is needed to elucidate this mechanism.

NET-derived histones may contribute to the late phase of lung injury by recruiting activated neutrophils and by inducing them to produce proinflammatory cytokines and chemokines. A recent report showed that, among all distant organs, the histone content was highest in the lungs of mice after renal IR^10^. This may indicate that the lungs are the first organ to encounter histones released from the IR-injured kidney via the inferior vena cava. Due to the extensive surface area of their capillary bed, the lungs can easily trap these histones. Furthermore, the abundant NET formation described in this study may reflect an enriched reticuloendothelial system that contains numerous antigen-presenting cells in the lung.

A previous study using a quartz crystal microbalance twin sensor system showed that rTM had a high affinity for histones. The authors suggested that rTM inhibited histone-induced platelet aggregation by binding to histones^17^. Based on this, we stretched to the hypothesis that rTM might attenuate the distant lung injury that results from ischemic AKI by neutralizing the histones. Notably, pretreatment with rTM significantly attenuated the elevated MPO activity, the mRNA expression of inflammatory cytokines and chemokines, and the neutrophil count in the lungs after renal IR. We also demonstrated that rTM reduced the pulmonary histone content and NET formation augmented by ischemic AKI. Moreover, delayed treatment with rTM showed similar impact on the lungs after renal IR.

In other research, a protective effect of TM has been shown in ischemic AKI^16^. This result would indicate that the improvement in acute lung injury after treatment with rTM was caused by ameliorated IR injury in the kidneys. However, we showed that there was no significant difference in the degree of renal injury (i.e., plasma BUN level, tubular injury score, mRNA expression of inflammatory cytokines and NGAL) between the treated and untreated groups. Additionally, we found that administration of rTM had no significant impact on histone accumulation or NET formation in the IR-injured kidneys, which contained higher histone levels compared with the lungs. This implied that either the histones levels exceeded the neutralization capacity of rTM or that other immune effectors that are not targeted by rTM (e.g., reactive oxygen species and complements)^20^ contribute to local kidney injury after IR insults. Therefore, rTM therapy may directly benefit remote lung injury. Notably, IL-6 mRNA expression was significantly reduced in the IR-injured kidney by rTM administration (Fig. 3e). A previous report showed no improvement in renal function after ischemic AKI in IL-6-deficient mice or in wild-type mice treated with anti-IL-6 antibodies compared with controls^7^. Given these results, the contribution of IL-6 to the development of ischemic AKI may be modest.

Endothelial dysfunction is important to the pathogenesis of IR injury in local and remote organs. Tissue hypoxia impairs the integrity of endothelial adherens junctions via increased levels of inflammatory mediators such as thrombin, histamine, and vascular endothelium growth factor^21,22^. Positively charged extracellular histones also cause immediate endothelial cell death by binding to the negatively charged plasma membranes of endothelial cells and causing vascular leakage^23^. Significant extravasation of EBD was observed not only in the kidneys but also in the lungs after renal IR in our experiments, supporting the argument that circulating histones injure vascular endothelial cells (Fig. 4). Treatment with rTM markedly reduced this leakage of EBD in both the kidneys and the lungs, despite failing to decrease the histone levels in the IR-injured kidneys (Fig. 5b). Previous *in vitro* studies have shown that the lectin-like domain of rTM plays a pivotal role in maintaining the integrity of endothelial junctions^24^. In our study, rTM might directly protect cell-cell adhesion in the blood vessels of kidney and lung after renal IR in addition to neutralizing histones.

Recent animal studies have revealed that HMGB1 is an early mediator in IR injury of the liver, brain, heart, and kidney^25–28^, whereas it has been recognized as a late mediator in sepsis^9,29^. In a mouse renal IR model, HMGB1 was found to translocate rapidly from its normal site in the nucleus to the cytoplasm and out of cells by approximately 3 h after the ischemic insult^30^. Decreased kidney function can also increase the half-life of blood HMGB1^31^. When released, HMGB1 can activate nuclear factor kappa B and induce proinflammatory responses by interacting with pattern recognition receptors, such as TLR 4 and the receptor for advanced glycation end products^32,33^. In the current study, we showed significantly elevated plasma HMGB1 levels as early as 6 h after ischemic AKI that persisted to 24 h. Additionally, treatment with rTM significantly decreased HMGB1 levels at 24 h after surgery. This is consistent with the results of a previous report that described TM as binding HMGB1 via its lectin-like domain to have an anti-inflammatory effect^15^. Furthermore, we recently showed that the administration of anti-HMGB1 antibodies can inhibit the mRNA upregulation of HMGB1 downstream cytokines in the lungs after ischemic AKI^9^, and the present study found that this could be applicable to rTM. Therefore, HMGB1 inhibition by rTM may, at least partly, contribute to lung protection after renal IR by suppressing inflammatory cytokine production in the lungs. However, rTM did not reduce HMGB1 levels at 6 h after renal IR (Fig. 8), possibly because the excessive HMGB1 release from injured tubular cells overwhelmed the neutralizing capacity of rTM during the early phase. The different performances of extracellular histones and HMGB1 over time after renal IR suggests that different mechanisms contribute to distant lung injury in the early and late phases of reperfusion after ischemic AKI.

We have to acknowledge a limitation. Given rTM has several different targets, the results obtained in this study do not confirm a causal relationship between rTM and reduction of NETs and lung injury. Further assessment will be necessary using rTM preparations, which are pre-blocked at the binding site for histones.

In conclusion, rTM treatment is associated with suppression of distant lung injury that is characterized by neutrophil infiltration, elevated MPO activity, and inflammatory cytokine expression after renal IR. This treatment appears to act by blocking histone accumulation and NET formation in the lungs without ameliorating the IR-induced kidney injury. Treatment with rTM might be a potential strategy to attenuate the acute lung injury complicating AKI and improve the clinical outcomes of critically ill patients with AKI indirectly.

## Materials and Methods

### Animals and surgical protocol

Eight- to ten-week old male C57BL/6J mice were obtained from the Tokyo laboratory animal science (Tokyo, Japan) for use in all experiments. The mice were kept in a 12 h light/dark cycle with free access to diet and water. All experiments were conducted in accordance with the NIH Guide for the Care and Use of Laboratory Animals (US Department of Health and Human Services Public Health Services, National Institutes of Health, NIH Publication No. 86-23, 1985) and approved by institutional review board of the University of Tokyo.

Mice were anesthetized by intraperitoneal injection of a mixture of ketamine hydrochloride (Daiichi-Sankyo, Tokyo, Japan) and xylazine hydrochloride (Bayer, Leverkusen, Germany). All procedures were done on anesthetized mice with body temperatures maintained at 37 °C on a controlled heating table. Bilateral renal IR was conducted as previously described^34^. Briefly, the renal pedicles were identified via flank incisions and clamped for 30 min with small nontraumatic vascular clips (Muromachi Kikai, Tokyo, Japan). After clamp removal, we confirmed blood flow had been restored by observing the kidneys returning to their original color. Sham-operated mice underwent an identical procedure with no artery clamping. The intraperitoneal administration of rTM (Asahi Kasei Pharma, Tokyo, Japan) was done to the IR + rTM group, while the same amount of saline was given to the IR and sham-operated groups at 30 min before the surgical procedure. At 6 h and 24 h after surgery, mice were killed and their blood, lungs, and kidneys were harvested for further analyses. In another experiment, the mice were injected with rTM or the same amount of saline at 6 h after bilateral renal IR (delayed treatment). The organs were harvested after 24 h after surgery of renal IR.

### Histological examination of lung and kidney tissue

Lungs and kidneys were fixed with 10% formalin for 24 h and embedded in paraffin. Three-micrometer sections of the organs were stained with hematoxylin and eosin. Neutrophils were counted in 10 randomly selected non-overlapping fields at ×400 magnification in respective sections of each mouse lung. The numbers of neutrophils in each lung and individual animal were averaged. Tubular injury was scored in a blind manner, as previously reported^35^.

### Plasma BUN and cytokine measurement

BUN was measured using the urease indophenol method (Wako Pure Chemical Industries). An absorbance 96-well plate reader (Spectra MAX Plus; Molecular Devices, Sunnyvale, CA, USA) was used at a wavelength of 570 nm. The concentrations of IL-1β, IL-2, IL-6, TNF-α, and IL-10 were determined in the plasma, using custom V-plex assays (Meso scale discovery, Rockville, MD, USA) according to the manufacturer’s protocol. The plasma HMGB1 concentration was measured using an established enzyme-linked immunosorbent assay (ELISA) kit according to the manufacturer’s instructions (Fuso pharmaceutical, Osaka, Japan).

### Measurement of MPO activity

MPO activity was assessed using a Myeloperoxidase Fluorometric Detection Kit (Enzo Life Sciences, Farmingdale, NY, USA) and an fmax Fluorescence Microplate Reader (Molecular Devices), as described previously^9^. Results were normalized for protein content determined by a BCA protein assay (Pierce Biochemistry, Rockford, IL, USA).

### Vascular permeability assay using EBD

Vascular permeability was evaluated using EBD (Sigma-Aldrich, St Louis, MO, USA), as described previously^9^. Results are expressed as micrograms of EBD per gram of organ tissue (wet weight).

### Measurement of histone content in the kidneys, lungs, and plasma

Histone levels in the plasma and organ tissues were measured by a sandwich ELISA kit (Cell Death Detection ELISA^plus^; Sigma Aldrich, St. Louis, MO, USA). This assay used monoclonal mouse antibodies against single-strand and double-strand DNA and histones (H1, H2A, H2B, H3, and H4) to identify histone-associated DNA fragments. To assess the histone content, lung and kidney tissue specimens were weighed and mechanically homogenized using MicroSmash (TOMY, Tokyo, Japan) in 1 mL of lysis buffer (containing 4-Nonylphenyl-polyethylene glycol; Roche Diagnostics, IN, USA) attached to the kit, before being centrifuged at 8000 × *g* for 20 min. The histone content of the supernatant was quantified based on the manufacturer’s instructions. Results are represented as absorbance units per milligram of organ tissue (wet weight).

### RNA preparation and Real-time PCR assay

Total RNA was isolated from lung and kidney homogenates using Trizol (Invitrogen, Carlsbad, CA, USA). A High Capacity cDNA Reverse Transcription Kit (Applied Biosystems, Foster City, CA, USA) was used in accordance with the manufacturer’s protocol to synthesize complementary DNA from total RNA with random primers. The FAST SYBR Green master mix (Applied Biosystems) was used for quantitative PCR to measure mRNA levels of IL-6, KC, and NGAL. The primer-specific nucleotide sequences of IL-6, KC, and NGAL are shown in Supplementary Table S1. The transcription levels of TNF-α and 18s ribosomal RNA (18s rRNA) were examined using TaqMan Gene Expression Assays (Applied Biosystems). The TaqMan primers used for the assays were TNF-α (sample ID number Mm00443258_m1) and 18s rRNA (sample ID number 4310893E). All gene expression levels were normalized using 18s rRNA as a housekeeping gene. Amplification data were analyzed using ViiA 7 Software ver.1.2 (Applied Biosystems).

### Immunofluorescence assay

Lungs and kidneys were frozen in Tissue-Tek OCT compounds (Sakura, Tokyo, Japan) and cryosectioned at 5 µm thicknesses. The sections were fixed with cold acetone for 15 min and blocked with phosphate-buffered saline, 1% bovine serum albumin, and 1% goat serum albumin at room temperature for 1 h. Next, sections were incubated overnight at 4 °C with the following primary antibodies: 1 µg/mL rabbit anti-mouse CitH3 antibody (Abcam, Cambridge, UK) and 2 µg/mL rat anti-mouse Ly6G/C (neutrophil marker) antibody (Santa Cruz Biotechnology, Dallas, TX, USA). The sections were then washed and incubated with the following Alexa-conjugated secondary antibodies (Invitrogen): Alexa 488-goat anti-rabbit IgG (4 µg/mL) and Alexa 555-goat anti-rat IgG (4 µg/mL) at room temperature for 1 h. DNA was stained with 1 µg/mL TOPRO-3 (Invitrogen). Confocal images were acquired using a Leica TCS SP5 II microscope (Leica, Wetzlar, Germany). The percentages of CitH3-positive and Ly6G/C-positive areas in each field were calculated in 10 randomly selected non-overlapping fields from respective lung and kidney sections at ×400 magnification. The percentages of positive areas were averaged in respective organs and individual animals. The imaging analysis was conducted in a blinded manner using MCID software (MCIC, Linton, Cambridge, UK).

### Statistical analysis

Differences between groups were analyzed for statistical significance by Student’s *t*-tests or by one-way analysis of variance followed by the Tukey–Kramer test for multiple pairwise comparisons. A two-tailed p value of <0.05 was considered statistically significant. All statistical analyses were conducted using JMP 13.0 software (SAS Institute, Cary, NC, USA). Data are expressed as means ± standard deviations.

## Supplementary information

Supplementary Information.

## Data Availability

The datasets analyzed during the current study are available from the corresponding author on reasonable request.
