# Supplementary material for: Recombinant thrombomodulin prevents acute lung injury induced by renal ischemia-reperfusion injury
Source: Sci Rep. 2020 Jan 14;10:289. doi: 10.1038/s41598-019-57205-0 (PMC6959219; doi:10.1038/s41598-019-57205-0)
Supplement: Supplementary file 1 — Supplementary Information. [file 41598_2019_57205_MOESM1_ESM.pdf]

Recombinant thrombomodulin prevents acute lung injury induced by renal ischemia-reperfusion injury

Naoki Hayase, Kent Doi, Takahiro Hiruma, Ryo Matsuura, Yoshifumi Hamasaki, Eisei Noiri, Masaomi Nangaku, and Naoto Morimura

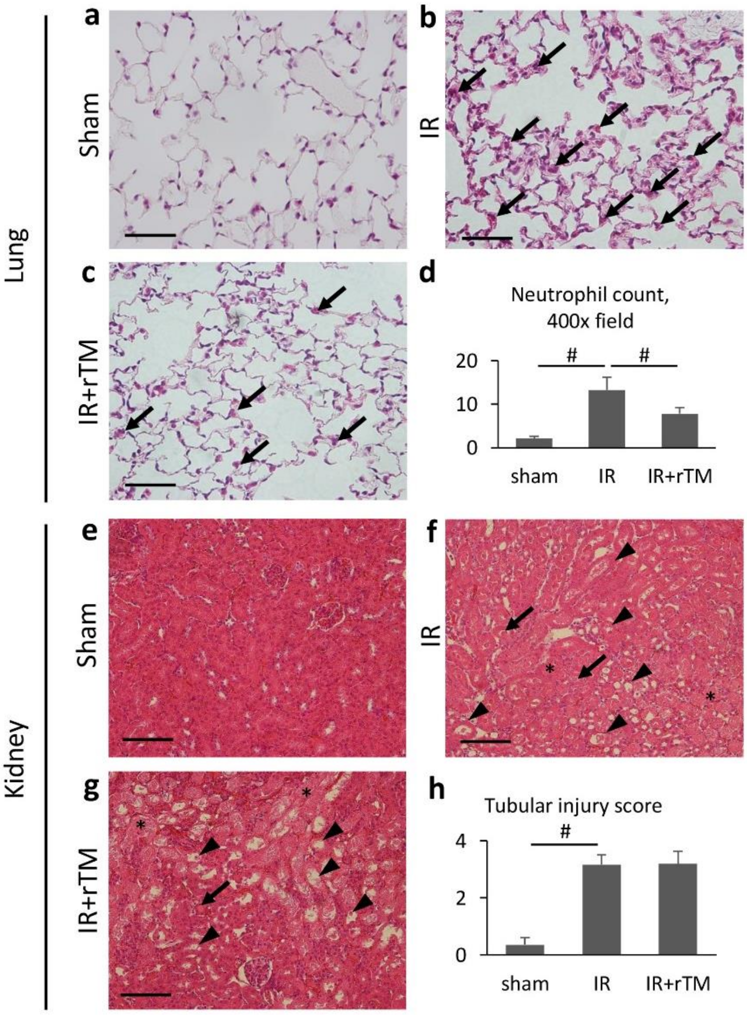

Supplementary Figure S1. Pulmonary and renal histology of renal IR-injured mice (delayed treatment group)

Renal IR model mice (30-min ischemia followed by 24 hours of reperfusion) were treated with saline or 10 mg/kg rTM at 6 h after surgery. Representative images of lung sections (hematoxylin and eosin staining) from (a) the sham-operated, (b) IR, and (c) IR + rTM groups are shown. Scale bar = 50 µm. Remarkable neutrophil infiltration (arrows) was observed in the lung after renal IR. (d) The number of neutrophils in the lung after surgery (n = 5 per group). Representative image of renal sections (hematoxylin and eosin staining) from (e) the sham-operated, (f) IR, and (g) IR + rTM groups are shown. Scale bar = 50 µm. Arrows (f and g) denote cast formation, and arrowheads (f and g) exhibit tubular dilation and loss of brush border. Asterisks (f and g) show cell lysis. (h) Average score of tubular injury for respective animals was determined. n = 5 per group. # $P < 0.05$ versus respective control. Abbreviations: IR, ischemia-reperfusion; rTM, recombinant thrombomodulin.

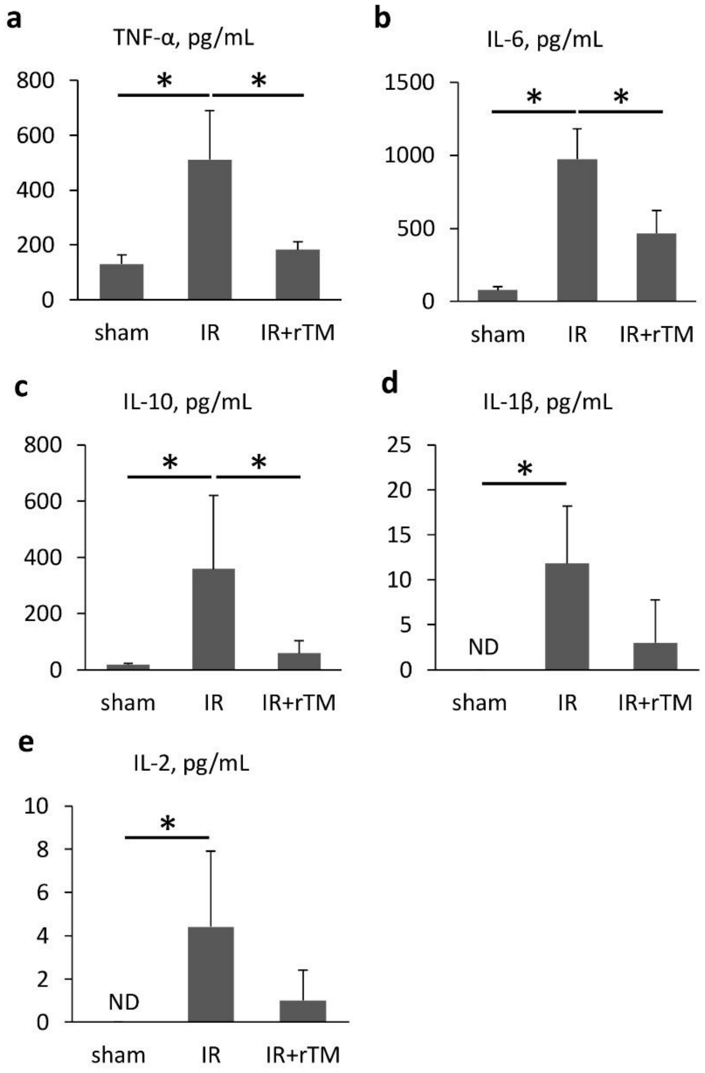

Supplementary Figure S2. The plasma levels of inflammatory and anti-inflammatory cytokines after renal IR

A total of 5 plasma cytokines including (a) TNF-α, (b) IL-6, (c) IL-10, (d) IL-1β, and (e) IL-2 were measured at 24 h after bilateral renal IR. The mice were pretreated with saline (IR group) or 10 mg/kg of rTM (IR + rTM group). n = 3 in sham group; n = 5 in IR and IR + rTM groups. * $P < 0.05$ versus respective control. Abbreviations: IL, interleukin; IR, ischemia-reperfusion; ND, not detected; rTM, recombinant thrombomodulin; TNF-α, tumor necrosis factor-α.

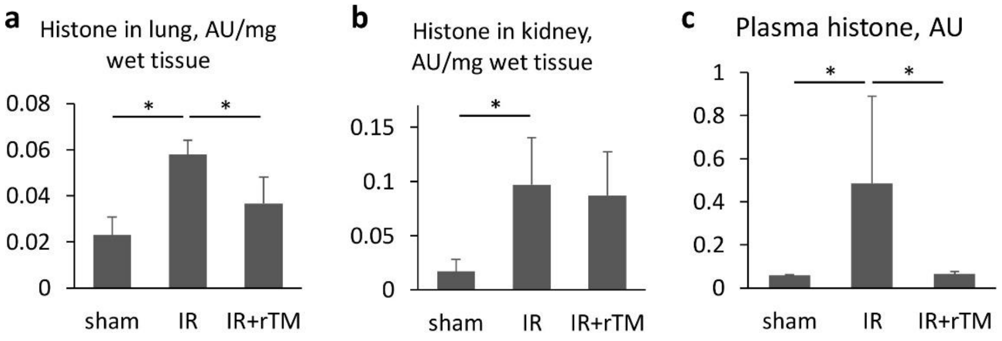

Supplementary Figure S3. Accumulation of histones in the lung and kidney after renal IR (delayed treatment group)

Histone levels in (a) the lung, (b) kidney, (c) plasma were evaluated after 30-min ischemia followed by 24-h reperfusion. The mice were injected with 10 mg/kg of rTM or vehicle solution at 6 h after surgery. n = 6 per group. * $P < 0.05$ versus respective control. Abbreviations: AU, absorbance unit; IR, ischemia-reperfusion; rTM, recombinant thrombomodulin.

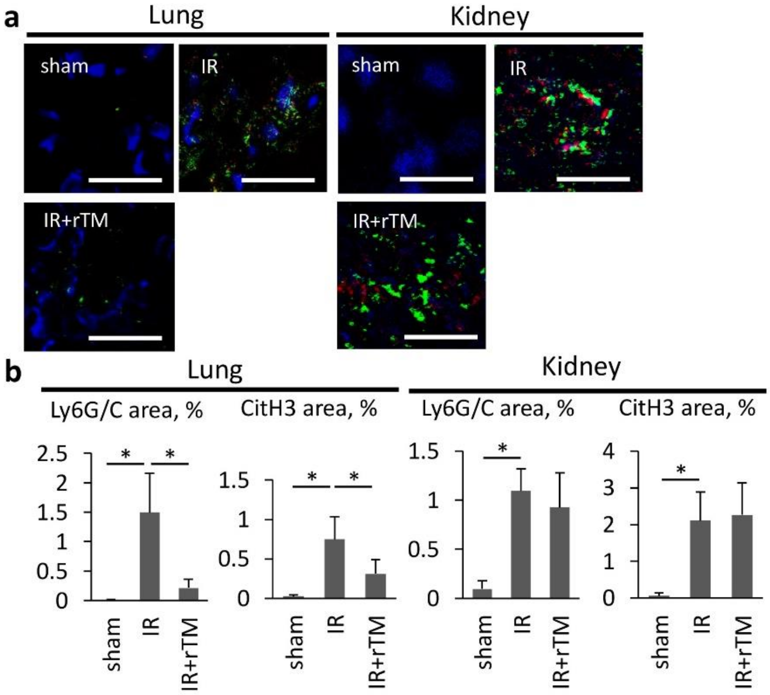

Supplementary Figure S4. NET formation in the lungs and kidneys after renal IR (delayed treatment group)

Representative images of NETs in the lung and kidney are shown with positive staining for CitH3 (green), Ly6G/C (red), and DNA (blue). The impact of delayed rTM treatment (administration of 10 mg/kg rTM at 6 h after renal IR) on NET formation in the lung and kidney were assessed by (a) an immunofluorescence assay and (b) quantitative analysis of NETs at 24 h after surgery. Scale bar, 25 µm. n = 7 per group. * $P < 0.05$ versus respective control. Abbreviations: CitH3, citrullinated histone H3; IR, ischemia-reperfusion; NET, neutrophil extracellular trap; rTM, recombinant thrombomodulin.

Supplementary Table S1. Primers used for real-time RT-PCR

Gene	Forward Primer Sequence	Reverse Primer Sequence
IL-6	TTCCATCCAGTTGCCTTCTT	ATTTCCACGATTTCCCAGAG
KC	GGCTGGGATTCACCTCAAGAAC	TGTGGCTATGACTTCGGTTTGG
NGAL	CCCTGTATGGAAGAACCAAGGA	CGGTGGGGACAGAGAAGATG

Abbreviations: *IL-6*, interleukin 6; *KC*, keratinocyte-derived chemokine; *NGAL*, neutrophil gelatinase-associated lipocalin; RT-PCR, reverse transcription polymerase chain reaction.
